# An abstract model of the basal ganglia, reward learning and action selection

**DOI:** 10.1186/1471-2202-12-S1-P189

**Published:** 2011-07-18

**Authors:** Pierre Berthet, Anders Lansner

**Affiliations:** 1 Department of Numerical Analysis and Computer Science, Stockholm University, Stockholm, 106 91, Sweden; 2 Stockholm Brain Institute, Karolinska Institutet Stockholm, 171 77, Sweden; 3 Department of Computational Biology, Royal Institute of Technology (KTH), Stockholm, 106 91, Sweden

## 

Learning is of major interest for various fields, from human pathologies to artificial intelligence. Animal and biology experiments have provided a great amount of data in this area, especially in classical and operant conditioning. Reinforcement learning is largely used in computational models in order to reproduce and explain these observations. This method enables to model a wide range of phenomena from the neuronal level to the behavior of a whole organism. Studying how information about reward or punishment is processed in the brain is crucial in the understanding of the action selection and decision-making in normal and pathological conditions. Basal Ganglia are a group of nuclei playing a major role in processing motor, associative and limbic information [1] and could be specialized in resolving conflicts between these sub-systems that compete for access to limited cognitive ressources [2]. Dopaminergic neurons activity is believed to be related to the reward prediction error and is involved in long term potentiation (LTP) and depression (LTD) in the striatum [3].

We present here an abstract computational model of the Basal Ganglia using reinforcement learning and Bayesian inference. It is based on a dual pathway architecture similar to the direct / Go and indirect / NoGo pathways that have been found in biology [4]. The units could be seen as grandmother cells, with inputs consisting of states and outputs being actions. Thus as a result of the activity in both pathways, an action will be selected based on the state and the previous outcomes of the different actions when dealing with the same state, that is if a reward has been obtained or not. One aim of our model is to be biologically plausible, weights can be updated via a triple activation Hebbian learning rule, similar to pre-synaptic activity, post-synaptic depolarisation and dopamine level [5]. The update equation is based on the probability of co-activity of the different active units. In its current form, the model uses trace activation and a simple delay mechanism. Basically, when a reward occurs, the weight between the active units is increased in the Go projection while it is decreased in the NoGo connection.

In a one to one mapping learning scheme (only one specific action for a given state triggers a reward), the simulation shows good results in both learning and re-learning, i.e. the mapping is then shuffled.

One interesting feature is the homeostasis property of the units: the variations of the sum of the outgoing and ingoing weights and bias, of a particular unit, are very small. In constrained set up (low numbers of possible actions and states), similar to experimental design, learning in stochastic reward occurrence is handled and the results are similar to the data. The response of midbrain dopamine neurons is positively correlated with the number of unrewarded trials [6] and our model produces a similar result with the prediction-error value. Future works will focus on reproducing conditioning phenomena and implementing spiking neurons.
